# Socioeconomic factors associated with drug consumption in prison population in Mexico

**DOI:** 10.1186/1471-2458-12-33

**Published:** 2012-01-13

**Authors:** Armando Nevárez-Sida, Patricia Constantino-Casas, Angélica Castro-Ríos

**Affiliations:** 1 Research Unit in Health Economics, Mexican Institute of Social Security (IMSS), Av. Cuauhtemoc # 330, Zip Code 06720 Mexico City, Mexico; 2 Coordination of Medical Units of High Specialty, Mexican Institute of Social Security (IMSS), Durango # 289, Zip Code 06700 Mexico City, Mexico; 3 Research Unit in Epidemiology and Health Services, Mexican Institute of Social Security (IMSS), Av. Cuauhtemoc # 330, Zip Code 06720 Mexico City, Mexico

**Keywords:** Illicit Drugs, Prison, Socioeconomic Factors, Family Characteristics, Mexico

## Abstract

**Background:**

Consumption of illegal drugs is a public health problem in Mexico, and the prison population is a vulnerable group with higher rates of prevalence than in the general population. The objective of this study was to determine the main socioeconomic variables associated with drug consumption in the prison population.

**Methods:**

Utilizing data from the Second Incarcerated Population Survey carried out by the Centre of Research and Teaching of Economics (CIDE) in Mexico, a logistic model in two stages was developed. The first stage analyzed the determinants of habitual drug consumption by prisoners (prior to admittance into prisons), while the second stage of the model addressed drug consumption within prisons.

**Results:**

Prevalence of drug consumption previous to incarceration was 28.5%, although once people were imprisoned this figure dropped to 7.4%. The characteristics that most heavily influenced against the possibility of habitual drug consumption prior to admittance to prison were: preparatory school or higher, being employed and having children; while the variables associated negatively were: male gender, childhood home shared with adults who consumed illegal drugs; abandoning childhood home; and having previous prison sentences. Once in prison, the negative conditions in there are associated with drug consumption.

**Conclusions:**

Work and study during incarceration, in addition to being instruments for rehabilitation, seem to exert an important positive association against drug consumption. However, this correlation seems to be minimized in the face of negative conditions of the penal institution; thus, public policies are necessary to improve the prisoner's environment.

## Background

Worldwide, Mexico is one of the main producers of illicit drugs, and during recent decades it has also become an important consumer, mainly due to its condition as a passageway to supply markets in the United States of America.

These illegal substances, consumed for their mind-altering proprieties, often generate dependence and, in some cases, in the long term, cause medical conditions such as neurological, cardiovascular, respiratory, hepatic and metabolic complications [1].

In Mexico, the consumption of illegal drugs increased from 4.6% in 2002 to 5.2% in 2008; the consumption of marijuana was 4.2% and of cocaine 2.4% [2]. The age of initiation of consumption is on average 19 years in males and 23 years in females [3].

Populations with disadvantaged socioeconomic characteristics are more vulnerable in relation to drug consumption. Among the principal risk factors reported in connection with drug consumption are: low educational levels, high rates of unemployment, dysfunctional families, and psychological and psychiatric problems [4].

Particularly, unemployment and drug consumption become linked into a vicious cycle. On the one hand, unemployment is a significant risk factor in drug consumption and it increases the possibility of relapse after rehabilitation following alcohol and drug addictions. On the other hand, illegal drug consumption increases the likelihood of unemployment and decreases the possibility of finding and keeping a job [5].

The incarcerated population combines many of those risk factors involved in drug consumption. Besides that, the length of prison sentence and the negative internal conditions of the penal institutions aggravate the situation.

In Mexico City and the adjoining State of Mexico 25% of the total prisoners in Mexico are concentrated, and, according to penal authorities in these geographical areas, the prevalence of drug and alcohol consumption is above 40% [6].

Regarding health considerations, attention should not only be paid to the damage caused by drug consumption but also to its indirect consequences. Users of illegal drugs, such as heroin or cocaine, have a greater risk of catching infectious diseases such as HIV/AIDS and hepatitis, as well as tuberculosis and other sexually transmitted diseases [7].

This risk of infection could be higher for the prison population if the internal conditions of the penal institutions increase the availability of those kinds of drugs. Some related evidence has been reported among HIV-positive prisoners where 85% of them were already infected when they arrived to prison. That means that 15% of HIV-positive prisoners were infected during incarceration; the same study reported that 90% of prisoners were users of injectable drugs [8].

The objective of the present study was to determine the main socioeconomic variables associated with drug consumption prior to incarceration and once inside these institutions, in the prisoner population of Mexico City and the State of Mexico.

## Methods

We utilized the Second Survey of the prison population in Mexico City and the State of Mexico carried out by the Centre of Research and Teaching of Economics (CIDE). Information from this survey is available openly in http://www.cide.edu.

The survey includes information related to sociodemographic characteristics, trends of illegal activities, conditions of life in prison and the efficiency of legal process. This survey was conducted at the end of 2005 and the beginning of 2006; the large penal institutions and a sub-sample of small penal centres were included as a sampling frame with a universe of 49,633 prisoners. The sample was selected by systematic sampling with a randomized start and included 1,223 persons. First, the number of surveys to be applied was distributed according the total number of prisoners at each institution to reach the pre-defined quota [9].

Participation in the survey was voluntary. The prison authorities provided a private setting in which to carry out the surveys, ensuring total confidentiality.

For descriptive statistical analysis [10], we used Chi^2 ^and Pearson tests for binary variables, the Fisher exact test for categorical variables, and the Student *t *test for continuous data. Similarly, we carried out one- and two-way analysis of variance and the post-hoc Bonferroni test for comparison among groups, with a significance level of 0.05.

### Description of the regression model

A logistic regression model of the probability of drug consumption in the prison population was developed. The model had two stages, the first one estimated the probability of habitual drug consumption before being admitted to the penal centre, and it took into account current sociodemographic characteristics and also those in the prisoner's childhood. The second model's stage evaluated drug consumption while being in prison, it considered as explicative variables the characteristics of the prison, the crime and associated sentence, as well as the probability of habitual drug consumption before the current incarceration (results of stage 1).

Illegal drug consumption was defined combining the type of drug consumed and the frequency of consumption. The drugs considered as illegal were: heroin, marijuana, crack, cocaine, pills and inhalants (glue and cement).

The dependent variable of the first stage of the model was "habitual illegal drug consumption" and it was defined according to the consumption of one or more illegal drugs and using them at least once a week during 6 months prior to detention. This variable was dichotomous, in which the value of 1 was assigned to prisoners considered habitual drug consumers.

The dependent variable for the second stage of the model was "Drug consumption during the current incarceration" and was defined as to whether or not the prisoner reported having consumed one of the illegal drugs in the last month in prison. This variable was also dichotomous.

In the first stage of the model the independent variables included were the following:

General characteristics

a) Gender, assigning the value of 1 for males

b) Age, as a discrete variable expressed in years completed

c) Schooling, as a categorical variable with values of 1, 2, 3, and 4 for prisoners with incomplete elementary school, complete elementary school, high school, and bachelor school or higher, respectively

Childhood ambience

d) Marginalization at childhood home, as the index of marginalization of the geographical area in which the prisoner was born. This is calculated by Mexico's National Population Council (CONAPO) [11], considering the characteristics of the locality such as: educative level, living conditions, and the availability of services; the numerical magnitude has a direct relationship with the level of marginalization

e) Childhood home shared with adults with drug consumption: Assigning the value of 1 if one or both of the prisoner's parents or close adults who lived in the infancy or childhood home consumed illegal drugs

f) Abandoned childhood home: when the prisoner abandoned home due to economic or family problems when he/she was younger than15 years and remained outside his/her home for more than 1 month

Adulthood ambience

g) Employment prior incarceration: if the prisoner was employed prior to the current incarceration

h) Having a permanent partner: whether the prisoner reported being legally married or living with concubine

i) Having children: if he/she had children of his/her own

Prison record

j) Previous sentence: whether the prisoner had been sentenced before the current sentence

k) Criminal pattern: type of criminal activities that the prisoners reported defined as a categorical variable with values of 1, 2, and 3 for prisoners for crimes against persons (homicide, kidnapping, inflicting injuries, sexual crimes and robbery with violence), property (simple robbery, house breaking and entering and extortion), and harming health, respectively. Crimes harming health include any crime affecting health, ranging from consumption of an illegal drug to murder, usually related to illegal trafficking or consumption of drugs

For the second stage of the regression model, the independent variable was drug consumption by the prisoner during the last month. The following independent variables were considered:

a) Type of current crime for which the prisoner is completing the current sentence, a variable category with values of 1, 2, and 3; crimes against persons, property, and health (including trafficking of illegal drugs), respectively

b) Positive conditions during incarceration: defined as the devotion of the greater part of the prisoner's time to personal development activities, which promote re-adaptation, such as study or remunerative employment

c) Negative conditions in prison, assigning the value of 1 if the prisoner reported situations of abuse or discrimination in prison, such as being charged a fee for allowing family visits, telephone calls, receiving food, clothing, or other essential objects

d) The location of the prison was a dichotomous variable, with 1 corresponding to Mexico City and 0 to the State of Mexico

e) Months of the current sentence spent in prison, the number of months spent in prison due to the current sentence

f) Probability of habitual drug consumption prior to current incarceration corresponds to the estimated probability that a person would consume drugs prior to being in prison according to stage 1 of the model. This variable includes the effect of the variables of the model of Stage 1

The odds ratios of the probability of illegal drug consumption and its 95% confidence intervals were reported for each variable and each stage of the model. The odds ratios represent the change in the probability when compared with a reference group.

To evaluate logistic models, Hosmer-Lemeshow Goodness-of-fit tests and the odds of verisimilitude tests were applied to choose the final model. The possible effect of confusion or modification of the independent variables, such as employment prior to incarceration, and the positive and negative conditions of the penal institution were evaluated.

Since the probability of illegal drug consumption before entering the prison is a non-visible variable, and it was derived from stage 1 of the model, the sample error can be underestimated. Therefore, it is advisable to make an adjustment in order to obtain consistent coefficients and an asymptotic covariance matrix for the second-stage [12]. For this purpose we used the sandwich estimate [13]. Stata 11 software was used for statistical analysis.

## Results

The final total sample included 1,223 prisoners; it is noteworthy that 6% refused to participate, 2% of prisoners had to be replaced because they were in a detoxification programme, and an additional 2% were unable to respond to questionnaires due to their health conditions.

In the sample 82.5% were male, 11.2% had bachelor school degrees or higher, and 88% were employed. Regarding their family history, 4.2% shared a childhood home with adult with drug consumption, 8.7% had to abandon their childhood home before they were 15 years old, 86% had a current partner and 71% had children of their own.

About their prison record, 24.7% had a prior sentence, spending on average 34 months in prison. The most frequent type of crime was against persons (58.1%), 31.3% against property and 10.8% against health. On average, prisoners had spent 44.8% of their current sentence time.

Regarding penal conditions, 12.5% reported studying and 25.7% reported having remunerative employment. However, 48.1% reported negative conditions related to corruption and discrimination within the prisons.

Table 1 describes the characteristics of the survey prisoner population, comparing whether or not there was illegal drugs consumption, either prior to imprisonment (habitual) or during incarceration.

**Table 1:** General characteristics of the prison population

	Habitual			During imprisonment		
	Drug consumption		p	Drug consumption		p
Variables	Yes	No		Yes	No	
**General characteristics**	**28.5%**	**71.5%**		7.4%	92.6%	
Number of elements	348	875		90	1,133	
Gender - Male	89.1%	79.9%	*	86.7%	82.2%	
Age, mean (SD)	31 (7.6)	34 (9.8)	*	32.1 (7.7)	33.2 (9.5)	
Schooling						
Incomplete elementary school	15.0%	17.0%	*	14.9%	16.6%	*
Elementary school	51.9%	35.6%		55.2%	39.1%	
High school	27.7%	33.9%		26.4%	32.6%	
Bachelor school or higher	5.3%	13.5%		3.4%	11.7%	
**Childhood ambience**						
Index of marginalization at childhood home(SD)	-1.7 (0.6)	-1.4 (0.9)	*	-1.7 (0.5)	-1.47 (0.9)	*
Childhood home shared with adult with drug consumption	8.1%	2.7%	*	8.9%	3.9%	*
Abandoned childhood home	15.8%	5.9%	*	18.9%	7.9%	*
**Adulthood ambience**						
Employment prior incarceration	83.9%	89.5%	*	82.2%	88.4%	*
Having permanent partner	82.8%	86.9%		83.3%	85.9%	
Having children	58.3%	75.9%	*	65.6%	71.3%	
**Prison record**						
Prior sentence	42.5%	17.6%	*	50.0%	22.7%	*
Prior months in prison (SD)^a ^	37.7 (66.9)	33.1 (37.5)		54.4 (106.3)	32 (37.5)	*
Type of current crime						
Against persons	49.6%	61.5%	*	45.6%	59.1%	*
Against property	39.2%	28.1%		40.0%	30.6%	
Against health (Drug-related)	11.2%	10.4%		14.4%	10.3%	
Months in prison waiting for a sentence (SD)	8.5 (12.1)	10.4 (12.2)	*	7 (7.9)	10.1 (12.5)	*
Total months in prison (SD)	40.2 (36.0)	43.3 (35.4)		40.1 (40.5)	42.6 (35.2)	
Current sentence spent in prison	57.7%	39.7%	*	45.8%	43.2%	
**Penal institution characteristics**						
Location (Mexico city)	34.5%	65.5%	*	10.6%	89.4%	*
Location (State of Mexico)	22.0%	78.0%		3.9%	96.1%	
Positive conditions						
Study	12.9%	12.2%		11.1%	12.5%	
Remunerated employment	24.1%	26.3%		18.9%	26.2%	
Negative conditions	56.0%	45.0%	*	78.9%	45.7%	*
**Drug consumption**	**During imprisonment**			**Pre-imprisonment**		
Yes	21.8%	1.6%	*	84.4%	24.0%	*
No	78.2%	98.4%		15.6%	76.0%	

*Statistical significance < 5%, utilizing the chi^2 ^test for dichotomous variables and Student *t *test for continuous variables, supposing different variances

^a^For those with a prior sentence

The sociodemographic conditions and family history patterns seem to be worse in the groups involved in drug consumption; they reported a lower level of employment and schooling, and worse marginalization and childhood home ambience.

Prevalence of drug consumption prior to incarceration was 28.5%, although once imprisoned this figure dropped to 7.4%. Prisoners who reported consuming drugs within the penal institution also reported habitual consumption in the months prior to their imprisonment (84.4%), and the remainder (15.6%) began their consumption within the prison.

Drug consumption before imprisonment was: 72% marijuana; 27% inhalants, mainly glue and cement; 48% crack/cocaine; 27% pills and 6% heroin and other illegal drugs. Of these results, 51% reported only one type of drug, while 28% used two and the remaining 21% used 3 or more of the listed drugs.

Nevertheless, the pattern of drug consumption during imprisonment yielded somewhat different results: use of marijuana remained the same, but consumption of inhalants decreased to 5%, crack/cocaine to 15% and 11% pills. Also the number of drugs consumed simultaneously changed: 83% used only one drug, 16% two drugs, and the remaining 1% three or more combined.

Results are presented separately for each of the models. The first stage of the model analyses the consumption of drugs during 6 months prior to the prisoner's detention, and in the second stage of the model, drug consumption is evaluated while being in prison, during the last month.

### Stage I: Habitual drug consumption prior to incarceration

The results of the multivariate analysis for habitual drug consumption prior to entering prison are shown in Table 2. The association of socioeconomic factors and criminal behavior was analysed. Gender (female), age, and higher educative level are associated egatively with habitual drug consumption.

**Table 2:** Stage 1 of the regression model for habitual drug consumption prior to detention

Variables	Odds Ratio	95% Conf. Interval	
Gender (male)	2.859*	1.705	4.792
Age	0.978*	0.958	0.998
Schooling^a^			
Primary	1.462	0.936	2.281
Secondary	0.629	0.388	1.019
Preparatory or higher	0.473*	0.237	0.939
Index of marginalization at childhood home	0.700*	0.556	0.881
Childhood home shared with adults with drug consumption	2.685*	1.362	5.293
Abandoned childhood home	2.695*	1.620	4.482
Being employed prior to incarceration	0.524*	0.328	0.835
Having permanent partner	1.010	0.644	1.583
Having children	0.488*	0.337	0.706
Prior sentence	3.117*	2.211	4.391
Criminal pattern^b^			
Crime against persons	0.374	0.220	0.634
Crimes against property	0.353	0.199	0.624
Number of observations	1,032		
LR chi^2 ^(14)	197.77		
Prob >chi^2^	0.000		
Pseudo *R*^2^	0.1592		

*statistical significance < 5%

Reference categories. ^a ^Incomplete primary; ^b ^Drug-related crimes

The environment in which the prisoners spent their first years of life is represented by variables, such as index of marginalization at childhood home, having shared childhood home with adults who consumed drugs, and having abandoned childhood home at early ages. It was observed that the greater the marginalization in the childhood home, the less the possibility of habitual drug consumption; while having lived in infancy and childhood with adults who consumed drugs exerted a pernicious association with the possibility of using drugs when adult. And finally, family dysfunction, represented by the fact of having left home at an early age, is associated with a greater predisposition towards using drugs.

On the other hand, the family and social ambience prior to the prisoner's detention, that is, during the same lapse of drug consumption, shows that being employed had a positive association against habitual drug consumption, as did having children, while having a partner was not a significant variable for explaining habitual drug consumption.

Having previously been in prison is an important factor in explaining habitual drug consumption, as well as that of the criminal pattern for crimes against health, which implies a greater consumption possibility.

### Stage 2: Drug consumption during incarceration

In this model, the dependent variable is drug consumption in prison during the month prior to carrying out the survey. The type of crime for which the current sentence was imposed is not relevant for drug consumption in prison (Table 3).

**Table 3:** Stage 2 of the regression model for drug consumption during incarceration

Variables	Odds Ratio	95% Conf. Interval	
Positive conditions in prison	0.589*	0.346	1.00
Negative conditions in prison	3.654*	2.026	6.589
Months spent in prison of current sentence	1.006*	1.001	1.01
Type of current crime^b^			
Crimes against persons	0.486	0.234	1.008
Crimes against property	0.483	0.218	1.067
Prison location	1.332	0.735	2.410
Habitual drug consumption prior to current incarceration(stage 1)	29.877*	10.087	88.512
Number of observations	1,032		
LR chi^2^(7)	87.81		
Prob >ch^i2^	0.000		
Pseudo *R*^2^	0.156		

*statistical significance < 5%

Reference category: ^b ^Drug-related crimes

The time spent in prison increases the possibility for the prisoner to use drugs. The positive conditions in prison had a much lower association with drug consumption than the pernicious effect of the negative conditions.

The variable with the greatest association was the possibility of habitual drug consumption, which is provided by the estimates of the Stage 1 of the model, because previous consumption of drugs upon admittance into prison had an odds ratio of 29.8.

The location of the prison is not statistically significant in explaining drug consumption during incarceration.

## Discussion

This study evaluated the socioeconomic characteristics associated with drug consumption in the prison population in Mexico. The information, obtained in this study, is relevant to knowing the context in which drug consumption takes place in Mexican prisons. If more information is available about prisoners, it can be used by prison authorities in particular, by the government in general and by any person interested in developing programmes and policies aiming to prevent and reduce this problem. The results of surveys on the use and abuse of legal and illegal substances provide additional information to support prison personnel and enhance training in social and medical areas, especially psychiatry [14,15].

In this study, we found that the environment in which individuals spend their first years of life has key importance in the possibility of suffering problems with drug consumption in later life stages. The most relevant negative associations found were: having shared a childhood home with adults with drug consumption, and having abandoned a childhood home before being 15 years old. These results broaden the findings obtained by Negrete in a sample of Mexican students [16].

In this study, employment, a higher educative level, and having children, which could be a signal of appropriate entry into society, constitute the most relevant positive variables against drug consumption. In 1996, Bryant mentioned a negative impact of drugs on the propensity of persons to being employed [17]. The proportion of prisoners with some grade of upper education was lower than that reported for the general population; which was 14.5% in 2005 [18]. The marginalization in the childhood home had a negative association with habitual drug consumption, so maybe it could be related to the lower availability of illegal drugs in the childhood locality.

The effect of being witness to the drug consumption by adults when one is a child, and having lived outside the home at a very young age, had a negative effect on the possibility of using drugs, similar to having been in prison and devoting oneself to criminal activities related to crimes against health.

Criminal activity related to drugs importantly increases the possibility that the prisoner will have consumed drugs prior to his/her detention. However, once in prison, the association between the type of crime and drug consumption is not as relevant; that is, the negative impact of prison on drug consumption eliminates the differentiation that existed prior to going to prison for this type of crime, because the availability of these drugs is generalized in prison: 45% of the inmates that consume drugs do so on a daily basis.

The association of the prison conditions on drug consumption is very widespread; prisoners with more time to spend in prison have a greater possibility of consuming drugs; as the time of incarceration lengthens, the possibility of consumption of illegal drugs is greater.

Within the sample of prisoners in this study, the average time waiting for sentence was 10 months and that time in prison is a variable that is associated with drug consumption; it would be necessary to shorten the waiting time for sentencing, so that the innocent could gain freedom as soon as possible in order to decrease the possibility of succumbing to drug consumption.

In other studies, the prison-associated factors in the drug consumption problem, such as sentence type, the prison facilities, characteristics of the prison population and its personnel, as well as rehabilitation programmes, have been identified [19]. Likewise, psychosocial and psychiatric problems become intertwined with problems that are purely medical, and their importance should be taken into account in order to establish priorities for combating the consequences of drug consumption [20].

Life conditions in prison are difficult, since there is great overcrowding, insufficient and poor food and a lack of basic services, such as water for both drinking and personal hygiene. In this context, the positive association of helpful conditions of prison, such as work or study, has a much lesser impact than the prison's negative conditions on the possibility of consuming drugs.

Although in Mexico, there are education and work programmes in prison, they are not extensive due to the reduced capacity of these institutions, both in space, personnel and budgets. Inmate participation in the positive activities that do exist depends both on the prison conditions and the individual's interest, while the negative factors are related to a greater extent with corruption within the penal system. Thus, attacking this latter problem would have an important impact with respect to drug consumption by prisoners.

While the sample, utilized in the present study, includes the two main states in Mexico in terms of the size of the prison population, it is not possible to generalize the results obtained herein to the entire Mexican penal system, because of the heterogeneity that exists in the system as well as sociodemographic characteristics. It is important to conduct these types of studies with larger samples that represent the total population so that the implications for public policies, derived from these studies, are not limited to specific groups.

## Conclusions

The conditions that most frequently are associated with habitual drug consumption are: having lived during infancy or childhood with adults who consumed drugs and having abandoned home at early ages due to family problems, and having previous prison sentences. The characteristics that most heavily influenced against the possibility of habitual drug consumption prior to admittance to prison were: preparatory school or higher, being employed and having children. Once in prison, the sentence's length, as well as the negative conditions of being there, importantly increase the possibility of drug consumption during incarceration.

## Competing interests

The authors declare that they have no competing interests.

## Authors' contributions

ANS developed the concept and design for this paper. ANS and ACR have conducted the analysis and interpretation of data. ANS drafted the first version of the paper. PCC contributed to the critical interpretation of the data. All authors have been involved in revising the critiques of manuscripts and have read as well as approved the final manuscript.

## Pre-publication history

The pre-publication history for this paper can be accessed here:

http://www.biomedcentral.com/1471-2458/12/33/prepub

## References

[B1] Devlin RJ Henry JA Clinical review: major consequences of illicit drug consumption Crit Care 2008 12 1 202 208 10.1186/cc6166 18279535 PMC2374627

[B2] Secretaría de Salud Encuesta Nacional de adicciones 2008. México 2008 [Spanish]

[B3] Villatoro J Medina-Mora ME Cravioto P Fleiz C Galván F Rojas E Kuri P Ruiz C Castrejón J Velez A García A Encuesta Nacional de Adicciones. Instituto Nacional de Estadística, Geografía e Informática 2002 México: INEGI [Spanish]

[B4] Brooke D Taylor C Gunn J Maden A Substance misuse as a marker of vulnerability among male prisoners on remand Br J Psychiatry 2000 177 248 251 10.1192/bjp.177.3.248 11040886

[B5] Henkel D Unemployment and substance use: a review of the literature (1990-2010) Curr Drug Abuse Rev 2011 4 1 4 27 21466502 10.2174/1874473711104010004

[B6] Azaola E Bergman M De mal en peor: las condiciones de vida en las cárceles mexicanas Nueva Sociedad 2007 208 118 127 [Spanish]

[B7] Abdala N White E Toussova OV Krasnoselskikh TV Verevochkin S Kozlov AP Heimer R Comparing sexual risks and patterns of alcohol and drug use between injection drug users (IDUs) and non-IDUs who report sexual partnerships with IDUs in St. Petersburg, Rusia BMC Publ Health 2010 10 67 10.1186/1471-2458-10-67 PMC2988741 21054855

[B8] Hernandez R Coloooini R Idoate GA Scutze E Drug-addiction and AIDS in prison population Int Conf AIDS 1996 11 7 11

[B9] Hernández Sampieri R Fernández-Collado C Baptista Lucio P Metodología de la Investigación 2006 4 México: McGraw-Hill Interamericana [Spanish]11562851

[B10] Vittinghoff E Glidden DV Shiboski SC McCulloch CE Regression methods in biostatistics: linear, logistic, survival, and repeated measures models 2005 México, D.F. Springer

[B11] Consejo Nacional de Población CONAPO Índice de marginación a nivel localidad 2005 2007 México, D.F ISBN:970-628-924-0. [Spanish]

[B12] Murphy KM Topel RH Estimation and inference in two-step econometric models. (Statistical Data Included) J Bus Econ Stat 2002 20 1 88 97 10.1198/073500102753410417

[B13] Hardin JW The robust variance estimator for two-stage models Stata J 2002 2 3 253 366

[B14] Obradovic I Addictions in prison. A survey on socio-sanitary care for addicted prisoners using or overusing licit or illicit substances Tendances 2005 41 Available online at: [http://www.ofdt.fr/BDD/publications/docs/eftaiol1.pdf]: Sep 01 2010

[B15] Moreno JMP Psychosocial intervention with drug addicts in prison. Description and results of a programme Psychology in Spain 2000 4 1 64 74

[B16] Díaz-Negrete B García-Aurrecoechea R Factores psicosociales de riesgo de consumo de drogas ilícitas en una muestra de estudiantes mexicanos de educación media Rev Panam Salud Publica 2008 24 4 223 232 [Spanish] 19133170

[B17] Bryant RR Jayawardhana A Samaranayake VA Wilhite A The Impact of Alcohol and Drug Use on Employment: A Labor Market Study Using the National Longitudinal Survey of Youth 1996 University of Wisconsin Institute for research on poverty. Discussion paper no 1092 96

[B18] Instituto Nacional de Estadística Geografía e Informática INEGI II Conteo de Población y Vivienda 2005 2008 Instituto Nacional de Estadística Geografía e Informática ISBN 978-970-13-4999-1. [Spanish]

[B19] Shewan D Gemmell M Davies JB Prison as a modifier of drug using behaviour Addict Res Theory 1994 2 2 203 215 10.3109/16066359409109144

[B20] Castelli D Medical problems deriving from drug addiction in prison environment 1997 Sweiis Conference of Penitentiary Medicine, Switzerland Available online at: [http://www.aids-info.ch/files/pdf_files/symp_castelli_e.pdf]: Jul 03 2009

